# TOAST: a novel tool for designing targeted gene amplicons and an optimised set of primers for high-throughput sequencing in tuberculosis genomic studies

**DOI:** 10.1186/s12864-025-12247-9

**Published:** 2025-11-19

**Authors:** Linfeng Wang, Naphatcha Thawong, Joseph Thorpe, Matthew Higgins, Mark Tan Kia Ik, Waritta Sawaengdee, Surakameth Mahasirimongkol, João Perdigão, Susana Campino, Taane G. Clark, Jody E. Phelan

**Affiliations:** 1 Department of Infection Biology, Faculty of Infectious and Tropical Diseases, London School of Hygiene & Tropical Medicine, Keppel Street, London, WC1E 7HT UK; 2 Medical Life Sciences Institute, Department of Medical Sciences, Ministry of Public Health, Nonthaburi, Thailand; 3 iMed.ULisboa-Institute for Medicines Research, Faculty of Pharmacy, University of Lisbon, Lisbon, 1649004 Portugal; 4 Faculty of Epidemiology and Population Health, London School of Hygiene & Tropical Medicine, London, WC1E 7HT UK

**Keywords:** Amplicon sequencing, Amplicon design, Mycobacterium tuberculosis, Tuberculosis, Sequencing, Genomics

## Abstract

**Background:**

Amplicon sequencing of *Mycobacterium tuberculosis* resistance-associated genes offers a cost-effective alternative to whole-genome sequencing for rapid profiling of infections and guiding clinical management. However, existing assays require frequent manual updates to accommodate emerging resistance mutations, limiting scalability and responsiveness.

**Results:**

We present TOAST (Tuberculosis Optimised Amplicon Sequencing Tool), a novel software tool that automates primer design by integrating mutation frequencies from a curated database of over 68,000 drug-resistant *M. tuberculosis* genomes. TOAST prioritises regions with the highest clinical relevance, accounting for single-nucleotide polymorphisms, insertions, and deletions. The software supports customisation of design parameters such as amplicon length, melting temperature, and GC content, while screening for undesirable primer properties, including self-dimers and off-target binding. Using TOAST, we designed a multiplex panel of 33 amplicons targeting mutations associated with resistance to 13 anti-TB drugs. These amplicons covered over 97% of resistance mutations in a 68 K isolate database and were validated using Oxford Nanopore sequencing of two clinical samples, achieving high uniform coverage with a minimum sequencing depth exceeding 50-fold across all targets.

**Conclusions:**

TOAST represents a major advancement in targeted TB sequencing by integrating large-scale clinical genomic data directly into assay design. This enables rapid, high-coverage, and adaptable amplicon sequencing, enhancing diagnostic precision and surveillance capabilities for drug-resistant TB. TOAST’s framework is also extensible to other pathogens, supporting broader applications in infectious disease genomics.

**Supplementary Information:**

The online version contains supplementary material available at 10.1186/s12864-025-12247-9.

## Background

Tuberculosis (TB), caused by *Mycobacterium tuberculosis* (*Mtb*), remains a significant global public health challenge, with an estimated 10.8 million new infections and 1.3 million deaths reported in 2023 [1]. The emergence of drug-resistant *Mtb* has further exacerbated this crisis. Worldwide, an estimated 3.2% of newly diagnosed TB cases and 16% of cases with prior treatments are resistant to rifampicin (RR-TB) or to rifampicin and isoniazid, referred to as multidrug-resistant TB (MDR-TB). Furthermore, a significant subset (6.2%) of these MDR-TB/RR-TB cases escalate to extensively drug-resistant TB (XDR-TB), showing additional resistance to fluoroquinolones and at least one Group A anti-TB drug, such as bedaquiline or linezolid [1]. Addressing the global health challenge of drug-resistant TB necessitates the development of rapid, accurate, and cost-effective diagnostic tools for *Mtb* detection and the subsequent identification of drug resistance.

Drug resistance in *Mtb* is primarily caused by mutations in genes encoding drug targets or enzymes involved in drug activation [2]. These mutations include single-nucleotide polymorphisms (SNPs), small insertions and deletions (indels), as well as large deletions [3]. Around 92 loci [4] in *Mtb* genome (total size 4.4 Mb; ~4,000 genes) have been linked to drug resistance across 13 anti-TB drugs [5]. Knowing the complete set of mutations driving drug resistance can inform diagnostic design [6]. Recent data from next-generation sequencing platforms have enabled the identification of circulating resistance mutations and the discovery of novel mutations through phenotypic-genotypic association analyses. Approaches such as genome-wide association studies, machine learning, and phylogenetic methods have played a pivotal role in these advancements [7–11]. Complementary informatics tools, such as TB-Profiler [5], have been developed to predict genotypic drug resistance from sequence data [7].

Whole-genome sequencing (WGS) of *Mtb* has gained traction for both clinical and epidemiological investigations, including through the implementation of Illumina and Oxford Nanopore Technologies (ONT) platforms. To improve cost-effectiveness for low-resource settings, it is possible to target a select number of genes (e.g., drug resistance loci) across many samples using an amplicon-based approach (Amp-Seq) on NGS platforms. Unlike WGS, Amp-Seq selectively amplifies target sequences using Polymerase Chain Reaction (PCR) before high-throughput sequencing. Amplicon primer design is a critical determinant of sequencing accuracy and efficiency, requiring standardised melting temperatures (Tm) to minimise non-specific binding and inefficient amplification, both of which can compromise the integrity of Amp-Seq. Further, structural considerations in primer design, such as avoiding homopolymers, hairpins, homodimers, and heterodimers, are crucial to the optimisation of Amp-Seq assays. Though existing amplicon design tools and other general-purpose solutions, facilitate primer design, they often lack adaptability and full automation in accommodating the rapidly evolving genetic landscape of *Mtb* and the emergence of new resistance mutations. Current amplicon design tools, such as PrimerJinn [12] and Olivar [13], focus more on primer design and require the user to input the desired regions or amplicons manually. As drug resistance mutations and lineage diversity continuously emerge and evolve, the regions of diagnostic and clinical interest inevitably shift. Consequently, amplicon-based assays require frequent updating to maintain comprehensive coverage and efficacy in tracking and diagnosing evolving forms of TB. Similarly, frequencies of mutations can differ between geographic regions, potentially leading to different priorities on which mutations to capture. This necessitates advanced tools capable of dynamically incorporating new data on mutation frequency and genetic variation to guide amplicon design to maximise diagnostic precision and effectiveness.

Here we introduce TOAST (Tuberculosis Optimised Amplicon Sequencing Tool), a software tool designed to overcome current challenges in amplicon primer design by uniquely leveraging mutation frequencies from a customisable database of *Mtb* mutations. Amplicon targets are primarily selected based on high-frequency mutations observed in a curated dataset of over 68 K clinical isolates, which provides complete coverage of all WHO-defined resistance mutations [4] with additional coverage in genes such as *alr*. Importantly, SNPs are not treated equally but are ranked according to their observed frequencies across the dataset, enabling TOAST to prioritise clinically relevant and commonly occurring variants. The mutation database is fully customisable, allowing users to incorporate their own variant lists or update the database as new surveillance data becomes available. In the design of primer sets, TOAST optimises primer parameters such as melting temperature (Tm) and systematically avoids problematic features, including homopolymers, hairpins, homodimers, heterodimers, and alternative bindings through an integrated pipeline. By harnessing mutation frequency data, TOAST enables continuous, data-driven amplicon design, supporting robust and automated detection of evolving resistance variants. We demonstrate the reliability and effectiveness of TOAST-designed primers using drug-resistant *Mtb* samples from Portugal. The software is publicly available (https://pypi.org/project/toast-amplicon/), with a user-friendly web-based version accessible at https://genomics.lshtm.ac.uk/webtoast/#/.

## Implementation

### Software overview

TOAST (Tuberculosis Optimised Amplicon Sequencing Tool) is a command-line program designed to generate and identify TB amplicons based on user-defined parameters, such as the number and size of amplicons, while accommodating predefined existing amplicons. An iterative mutation search algorithm systematically scans the default 68K clinical sample database enriched with drug resistant samples or a user-provided database [5] within a defined window size (amplicon size), positioning amplicons at locations with the highest priority scores (based on the frequency of the mutation observed across the 68 K samples). Once optimal positions are identified, primer design methodology is employed to design primers within an extended range of the selected windows, ensuring they meet predefined criteria for melting temperature, GC content, hairpin prevention, and homodimer formation. The resulting primers are then screened in an all-versus-all manner to check for heterodimer binding and alternative binding at unintended genomic locations (S1 Figure).

Using these principles, TOAST can also estimate the number of amplicons required to achieve near-complete mutation coverage (e.g., 99%) for a given amplicon size. The tool provides a detailed output, including primer sequences, positions, and genomic regions covered. Outputs can be visualized using tools such as the Interactive Genomic Viewer (IGV) [14] (S2 Figure) alongside mapped sequencing data files (e.g., bam format), enabling seamless integration and analysis. This automated and robust design pipeline streamlines TB amplicon sequencing, making it a versatile tool for targeted mutation analysis and genomic surveillance.

### 68 K Database

The database was constructed by downloading and processing 68,395 publicly available *Mtb* whole-genome short read sequencing datasets from the ENA. The mutations compiled in the database comprehensively cover all WHO‑defined resistance-associated mutations [4] and include additional variants, such as those in the *alr* gene.

### Amplicon design

An optional preliminary step in TOAST involves estimating the number of amplicons required to cover all mutations in the provided database using the TOAST “amplicon_no” function. This step generates a line plot showing diminishing returns of mutation coverage as the number of amplicons increases. Following this, the TOAST design function can be used to generate detailed amplicon designs. For gene-based (gb) amplicon design, users can define the amplicon size (-a) and specify genes that must be fully covered (-sg). Nearly all design process variables are user-adjustable, while mutation frequency-based (mb) runs require users to input the desired number of amplicons (-nn). To facilitate primer amplification quality checks via gel electrophoresis, TOAST offers the -seg function, which allows the design of varying-sized amplicons. This function accepts comma-separated inputs for minimum amplicon size, maximum amplicon size, step size, and the number of amplicons per step. The algorithm then identifies the optimal combination of amplicons to cover each specific gene with minimal overlap. The remaining amplicons are assigned to genomic regions based on mutation weighting, ordered from the largest to the smallest amplicon size. Lastly, TOAST also offers the functionality of designing on top of existing primers (-ud).

By default, TOAST assumes the use of Q5 High-Fidelity DNA Polymerase. However, design criteria such as ideal melting temperature (Tm), GC content, primer size, mononucleotide repeats, self/pair complementarity, and DNA concentration can be customised through a user-defined JSON file using the -set option. Primer design leverages Primer3 software, which calculates a penalty score for each candidate primer [15]. Together, TOAST determines the optimal primer pair for PCR by evaluating factors such as melting temperature, GC content, primer length, and self-complementarity.

Primers that fail to meet strict thresholds, such as maximum GC content or minimum melting temperature, are excluded. For the remaining primers, penalty scores are computed based on weighted deviations from optimal values, factoring in end stability and potential mispriming. Primer pairs are additionally penalised for complementarity and melting temperature mismatches. The pipeline further evaluates primer pairs for alternative genomic binding and homodimer formation, discarding pairs that fail these checks, prioritising those with the lowest penalty scores.

Out of these, a stringent testing for alternative binding is implemented. Each candidate window of the genome, along with its reverse complement, was compared to the input sequence using a pairwise similarity threshold (> 90%). For candidate matches, a base-pairing integrity check was applied to the terminal region, followed by thermodynamic filtering based on melting temperature (Tm ≥ 50 °C) and Gibbs free energy (ΔG ≤ − 10 kcal/mol).

To ensure robust primer placement, a modifiable extended region, by default set to one-sixth of the amplicon size is added to both ends of the amplicon. This buffer region maximises the chances of locating suitable primer binding sites while preserving the amplicon ranges determined by the iterative mutation search algorithm. Primer sequences may include degenerate base pairs to account for mutations within binding sites, as identified from the 68 K database.

The output includes a CSV file (Primer_design) containing primer details such as amplicon ID, primer ID, melting temperature, sequences, and genomic coordinates. A mutation inclusion report (mutation_inclusion) specifies which mutations from the default 68K database or the user-defined database are covered by each amplicon. TOAST also provides plotting functions to visualise amplicon distribution by gene, along with a comparison to reference amplicon designs if available. Additionally, a BED file is generated, detailing amplicon positions, actual amplicon coverage, and primer coordinates, which can be visualized using the IGV.

### Culture, DNA extraction and sequencing

All primers were combined into a single pool for the amplification. A PCR master mix was prepared to a final volume of 25 µl, comprising 5 µl of Q5 Reaction Buffer (1X), 0.5 µl of dNTPs, 5 µl of High GC Enhancer, 0.25 µl of Q5 enzyme. 0.1 µl of each primer, 1.5 µl of DNA sample. The rest is made up with miliQ water. The PCR cycling conditions were as follows: tubes were placed in a thermal cycler set to an initial denaturation step of 30 s at 98 °C, followed by 35 cycles of denaturation at 98 °C for 10 s, annealing at 58 °C for 40 s, and extension at 72 °C for 55 s. After the cycles, a further extension at 72 °C for 5 min was performed. After the PCR reaction, the products were checked with gel electrophoresis, confirming that the intended segments had been successfully generated (S3 Figure). Next, using this full set of primers (10 µM), we conducted simultaneous amplifications on two MDR Mtb sourced from TB clinical cases in Portugal (Portuguese_MDR) and Angola (Angola_MDR) with respective concentration of 61.4 ng/µL and 64.4 ng/µL as measured by Nanodrop [16]. The sample DNA were obtained from a bacterial culture. Genomic DNA was extracted from cultured *M. tuberculosis* isolates using commercial column-based kits (Qiagen, Hilden, Germany) according to the manufacturer’s instructions.

### Library preparation and amplicon sequencing

The amplicon products were quantified using a DS-11 FX + Spectrophotometer/Fluorometer (DeNovix) along with the Qubit™ 1X dsDNA High Sensitivity Assay Kit. Following the manufacturer’s protocol for ligation sequencing of amplicons, the amplicon library was prepared using the Native Barcoding Kit 96 V14 (SQK-NBD114.96, ONT). The prepared library was subsequently sequenced on the MinION Mk1B platform using R10.4.1 flow cells (ONT) and operated with MinKNOW v24.06.14 software (ONT). The Dorado baseballer (v0.9.1) was used to basecall the resulting data (model: dna_r10.4.1_e8.2_400bps_sup@v5.0.0).

## Results

### TOAST software

The tool, implemented in Python, is connected to a database comprising 68,395 *Mtb* isolates with whole-genome sequencing (WGS) data (the 68 K database) [8, 10]. This database represents the major *Mtb* lineages: L1 (3.1%), L2 (50.9%), L3 (5.0%), and L4 (36.0%). Among these, 10,230 (15.0%) exhibited genotypic HR-TB, 2,950 (4.3%) RR-TB, and 36,905 (54.0%) showed at least MDR-TB.

Critically, the size of the database allows for mutation profiling across 34 resistance-associated genes in relation to the their frequency in the database (S1 Table). In the 68K database, *katG* and *rpoB* key targets for isoniazid and rifampicin resistance detection-were among the most frequently mutated, with 40,002 and 43,364 total mutation events, respectively. Notably, *rpoB*, a gene known for its highly heterogeneous resistance landscape, exhibited the greatest mutational diversity with 1,007 distinct mutation sites occurring over 43,364 isolates. This highlights not only the breadth of sequence coverage but also the capacity of the database to reveal rare and previously underrepresented variants.

Genes mediating resistance to second line and companion drugs, such as *ethA* (11,814 mutations), *embB* (33,344), *fabG1* (14,208), *rrs* (17,791), and *rpsL* (22,979) were also featured prominently, underscoring the database’s utility for pan-resistance profiling. Moreover, the inclusion of lower-frequency events in genes like *ddn*, *thyX*, *fbiA*, and *ethR* extends the resolution of the database to capture potentially emerging resistance determinants.

### Amplicons and mutations covered

**Fig. 1:**
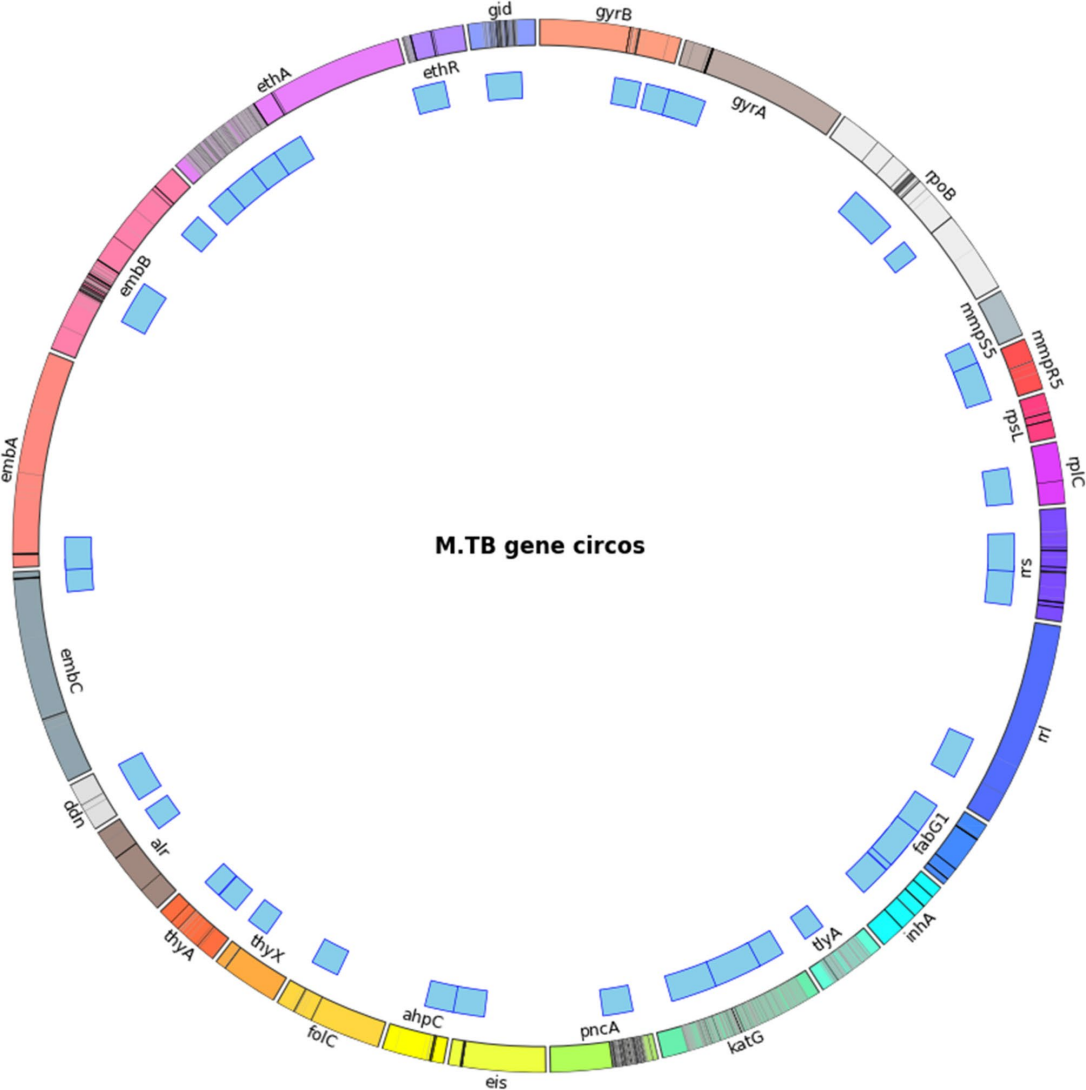
The 33 amplicons across the *Mtb* genome *Mtb* gene feature (multi-coloured outer ring) with mutation cytobands (more mutation with higher frequencies is in deeper colour) and amplicon position (Blue markers)

Using TOAST, thirty-three amplicon primers (forward and reverse pairs) were designed in 50 bp increments, ranging from 300 bp to 800 bp, with each increment repeated three times. These primers covered 2,230 known mutations across 25 genes associated with drug resistance, selected based on mutation frequency observed in the 68 K database (Fig. 1, S2 Table). The choice of 33 amplicons maximised mutation coverage, minimised diminishing returns on covering isolated rare mutations, and avoided primer clashes associated with higher numbers of amplicons (S4 Figure). This set of ONT-sized amplicons (500 bp), designed by TOAST, achieved 99.1% coverage of the established mutation loci (67743/68395). When tested with Illumina-sized amplicons (300 bp), the coverage reached 97.0%. On the other hand, longer amplicons (800 bp) suitable for ONT can also be generated, potentially expanding the flexibility of experimental designs and enabling the capture of more complex variants.

The amplicon coverage specifically included *katG* (isoniazid, 6 amplicons) and *mmpR5* (bedaquiline, 1 amplicon) in which loss of function resistance associated mutations can occur at any position. The remaining genes covered resistance to ethionamide (*ethA*, 4 amplicons), PAS (*folC*,* thyX*,* thyA*; 4), ethambutol (*embB*, 3), aminoglycosides/tuberactinomycins (*rrs*, 2), streptomycin (*rpsL*,* gid*; 2), linezolid (*rrl*,* rplC*; 2), rifampicin (*rpoB*, 2), fluoroquinolones (*gyrA*, *gyrB*, 2), capreomycin (*tlyA*, 1), D-cycloserine (*alr*, 1), kanamycin (*eis*, 1), pretomanid (*ddn*, 1) and pyrazinamide (*pncA*, 1). All drug resistance genes were completely covered by the designed amplicons, except for several loci with known localised genetic regions containing all or most of the known resistance-linked mutations: *gid* (99%), *rpoB* (98%), *katG* (92%), *embB* (92%), *ahpC* (92%), *inhA* (88%), *pncA* (72%), *embA* (50%), and *thyA* (29%). Importantly, the amplicons successfully captured most of the 68K database resistance mutations (2230/2506, 97.1%), with exceptions observed in *pncA* (155/166, 93.4%) for pyrazinamide resistance and *tlyA* (19/30, 63.3%) for capreomycin resistance (S3 Table). The *pncA* gene harbours a high number of low-frequency mutations and tends to receive lower coverage due to the weighting scheme based on sample frequency from the 68K database. However, the weighting scheme for mutation coverage can be adjusted to prioritise enhanced coverage in user-specified regions, providing- flexibility for targeted applications while enabling the development and implementation of locally adapted testing. This comprehensive amplicon design ensures robust coverage of known resistance loci, supporting detailed genomic surveillance and the detection of emerging drug-resistant mutations.

### Validation

**Fig. 2:**
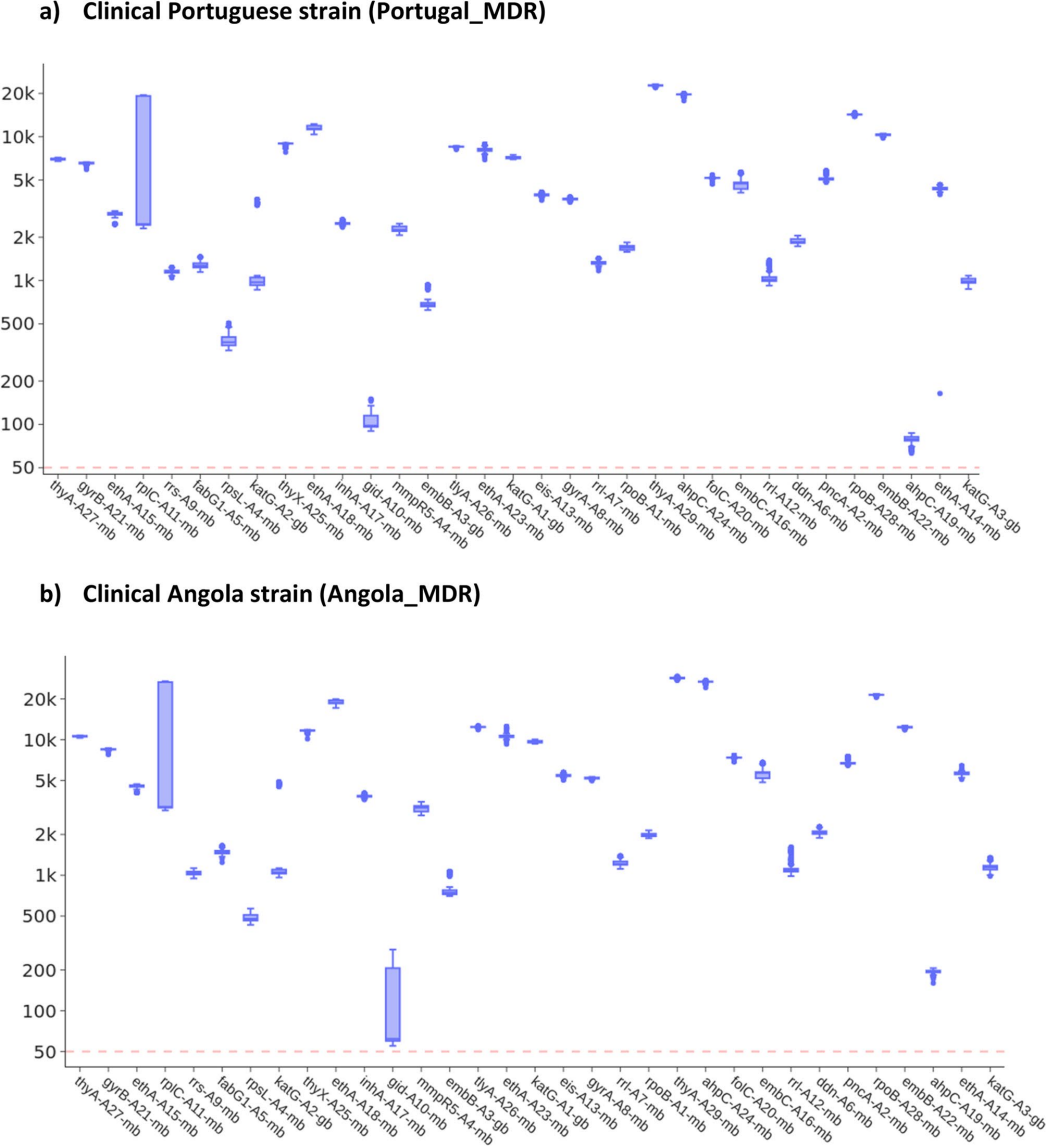
Boxplots of ONT sequence coverage depth for the 33 amplicons. **a** Clinical Portuguese strain (Portugal_MDR). **b **Clinical Angola strain (Angola_MDR). Dashed red line marks a 50-fold coverage threshold

The amplicons were validated through ONT sequencing of *Mtb* isolate DNA sourced from clinical MDR cases in Portugal (Portugal_MDR, 272439 reads; median length 401 bps) and Angola (Angola_MDR; 394977 reads; median length 406 bps). The vast majority of ONT reads mapped to the targeted 25 loci for both Portugal_MDR (261503/272439; 96.0%) and Angola_MDR (374609/394977; 94.8%). Most amplicon regions had high depth (>500-fold), except for *gid* (A10-mb) and *ahpC* (A19-mb) amplicons (Fig. 2). The minimum depth of coverage for *gid* A10-mb was 63-fold (Portugal_MDR 90-fold; Angola_MDR 63-fold) and for*ahpC* A19-mb was 55-fold (Portugal_MDR 55-fold; Angola_MDR 160-fold). The drug resistance profiles obtained from amplicon sequencing correspond perfectly to those obtained from WGS (16). Additionally, a subset of read samples was also performed at all 2-hour marks, with all amplicons showing >20-fold mean depth of coverage (S6 Figure).

The 33 amplicons ranged in size from 300 to 800 bp, with sequencing depth decreasing as amplicon size increased, likely due to reduced amplification efficiency (S5 Figure). Nevertheless, even amplicons exceeding 900 bp achieved a sequencing depth greater than 50-fold in our validation run (S5 Figure). While an inverse relationship between amplicon size and depth was observed, individual primer designs significantly influenced the resulting depth, occasionally causing substantial drops. This effect was more pronounced for shorter amplicons, whereas longer amplicons exhibited more consistent depth values.

## Discussion

The TOAST tool leverages an extensive and continually growing database of whole-genome sequencing (WGS) data, currently consisting of over 68,000 clinical *Mtb* isolates [5, 17]. This comprehensive mutation database not only guides the selection of high-value resistance targets but also informs the primer design process directly. In particular, common mutations within primer-binding regions are identified and accounted for, including through the incorporation of degenerate bases when necessary [18], ensuring robust amplification performance across lineages. As a result, TOAST enables precise and automated targeting of regions containing the most clinically relevant resistance mutations while maintaining compatibility across the global diversity of circulating strains.

TOAST provides both open-source availability and database-based mutation-informed design capabilities for automated amplicon design. TOAST is highly customisable, allowing users to define their own mutation lists and target loci, facilitating continuous updates to amplicon sets to include new or emerging resistance mutations without extensive reconfiguration.

We demonstrated TOAST’s utility by designing a multiplex assay consisting of 33 amplicons targeting critical drug resistance genes, comparable to previously proposed and commercially available amplicon sets [19].

Recent work has also explored nanopore-based targeted sequencing to address the limitations of whole-genome approaches for low-input or culture-derived DNA. Tang et al. [20] demonstrated that multiplex PCR coupled with ONT sequencing could rapidly detect resistance mutations across a reduced set of loci, validating feasibility in both cultured isolates and clinical samples.

Our TOAST-designed panel extends this concept by scaling up to a trial design of 33 amplicons that capture over 97% of known resistance mutations from a global 68 K isolate database, while automating primer design to accommodate emerging variants. By providing a framework for flexible, mutation-informed amplicon design, TOAST builds upon these advances to deliver a more comprehensive and adaptable assay that can be readily updated as new resistance mutations are identified.

Beyond TB, TOAST’s adaptable framework can be readily applied to other microbial pathogens by incorporating relevant genomic datasets and target regions. This versatility positions TOAST as a powerful resource in microbial genomics, enabling rapid response to the evolving threats and supporting improved surveillance, accurate diagnostics, and effective disease management, ultimately contributing to enhanced patient outcomes and strengthened global health efforts [8, 21].

## Conclusions

TOAST represents a major step forward in amplicon design by uniquely integrating a large, clinically derived database of over 68K *Mtb* genomes. This enables data-driven prioritisation of high-value resistance mutations, ensuring broad and clinically relevant coverage. Unlike existing tools, TOAST automates primer design based on real-world mutation frequencies, enhancing assay accuracy, flexibility, and adaptability to local epidemiology. Its robust performance in validation studies highlights its potential to improve TB diagnostics and genomic surveillance, while its extensible framework supports broader applications across microbial pathogens.

### Availability and requirements

Project name: TOAST.

Project home page: https://github.com/linfeng-wang/TOAST.

Operating system(s): Linux, MacOS.

Programming language: Python.

Other requirements: python 3.10 or higher.

License: MIT.

## Supplementary Information

Supplementary Material 1.

Supplementary Material 2.

## Data Availability

Raw sequencing data generated in this project is available from the ENA archive (accession numbers: PRJEB84199). The primers designed and tested in this study can be found in the supplementary data. The frequency data from the 68 K database and the full output of the test design can be found on author’s GitHub repository [https://github.com/linfeng-wang/TOAST]. The code for the software can be found on GitHub ([https://github.com/linfeng-wang/TOAST]. It is also available as a package ([https://pypi.org/project/toast-amplicon/].

## Abbreviations

TB: Tuberculosis
Mtb: Mycobacterium tuberculosis
Amp-Seq: Amplicon Sequencing
WGS: Whole Genome Sequencing
SNP: Single-nucleotide Polymorphism
indel: Insertion or Deletion
PCR: Polymerase Chain Reaction
Tm: Melting Temperature
GC: Guanine-Cytosine
ONT: Oxford Nanopore Technology
NGS: Next-Generation Sequencing
RR-TB: Rifampicin-Resistant Tuberculosis
MDR-TB: Multidrug-Resistant Tuberculosis
XDR-TB: Extensively Drug-Resistant Tuberculosis
HR-TB: Isoniazid-Resistant Tuberculosis
TOAST: Tuberculosis Optimised Amplicon Sequencing Tool
IGV: Integrative Genomics Viewer
PAS: Para-aminosalicylic acid
ENA: European Nucleotide Archive
