# Supplementary material for: TOAST: a novel tool for designing targeted gene amplicons and an optimised set of primers for high-throughput sequencing in tuberculosis genomic studies
Source: BMC Genomics. 2025 Nov 19;26:1058. doi: 10.1186/s12864-025-12247-9 (PMC12628886; doi:10.1186/s12864-025-12247-9)
Supplement: Supplementary file 2 — Supplementary Material 2. [file 12864_2025_12247_MOESM2_ESM.docx]

**Supplementary**

**S1 Figure**

**An overview of the TOAST workflow**


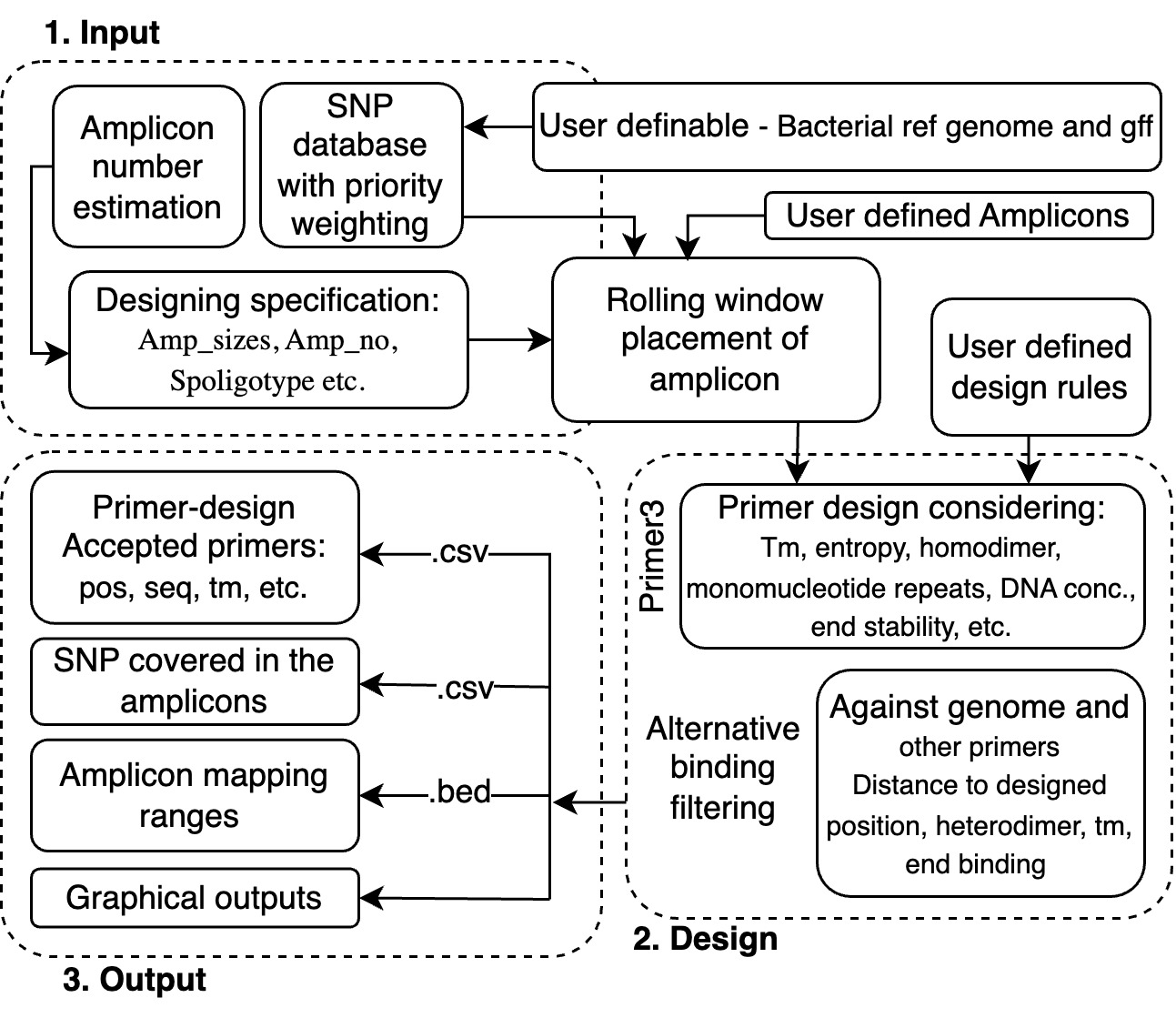
The TOAST workflow comprises three stages. In the input stage, mutation priority weighting (from a 68k dataset) and user-defined parameters guide amplicon placement using a rolling window approach. In the design stage, primers are generated using Primer3, considering factors such as Tm, entropy, and stability, followed by in-house filtering to eliminate alternative binding sites. The output stage provides primers with low penalty values and no alternative binding, along with SNP coverage details, amplicon mapping ranges, and graphical outputs.

**S2 Figure**

**IGV visualisation of designed amplicons**


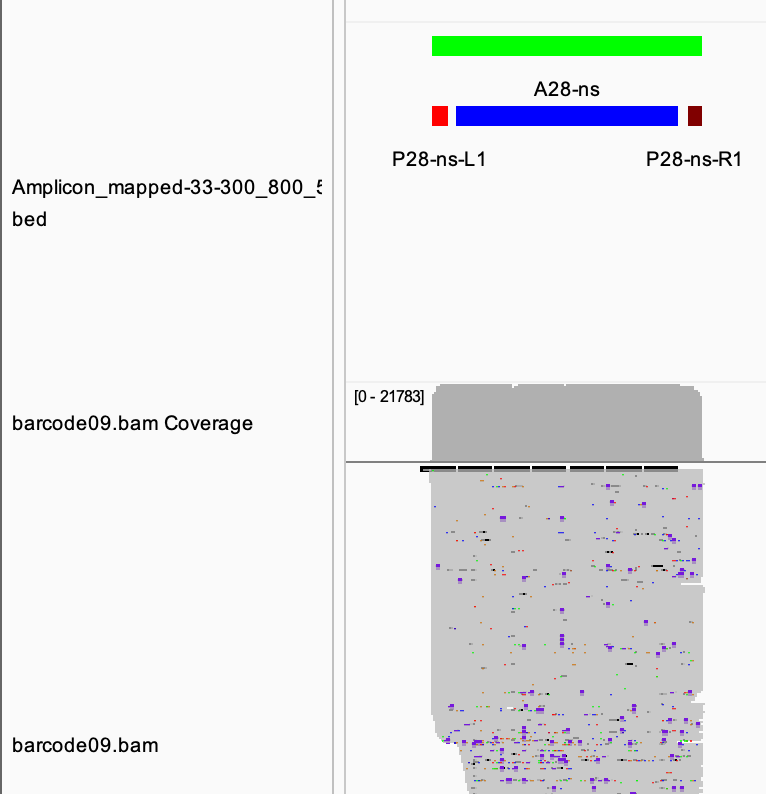


Red: left primer; brown: right primer; green: total amplicon size with buffer region and primer; blue: aimed range for amplification without buffer region and primer; grey: read coverage.

**S3 Figure**

**Gel electrophoresis photo for the 33 amplicon PCR.**

**
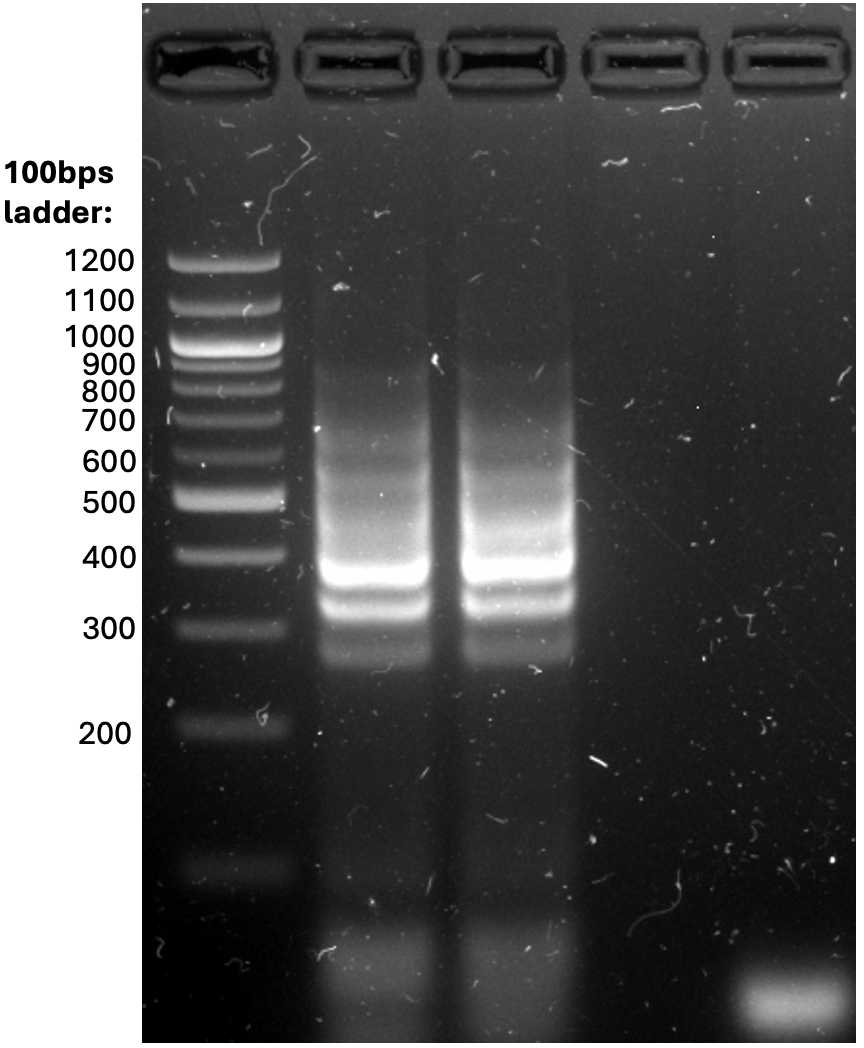
**

Lane 1

Lane 2

Lane 3

Lane 4

Lane 1: Annealing temperature at 58°C
Lane 2: Annealing temperature at 60°C
Lane 3: No load
Lane 4: Water (Negative control)

**S1 Table**

**50 dataset drug mutation coverage**

| **Gene** | **Mutation Count** | **Mutation Frequency** |
| --- | --- | --- |
| *rpoB* | 1007 | 43364 |
| *katG* | 862 | 40002 |
| *embB* | 757 | 33344 |
| *pncA* | 266 | 33251 |
| *rpsL* | 164 | 22979 |
| *rrs* | 136 | 17791 |
| *fabG1* | 101 | 14208 |
| *gyrA* | 26 | 14048 |
| *ethA* | 25 | 11814 |
| *gid* | 19 | 8620 |
| *eis* | 16 | 5907 |
| *inhA* | 16 | 4168 |
| *embA* | 16 | 3876 |
| *ahpC* | 15 | 1607 |
| *gyrB* | 12 | 1558 |
| *folC* | 12 | 1516 |
| *ethR* | 11 | 1425 |
| *thyX* | 10 | 1125 |
| *tlyA* | 10 | 1060 |
| *alr* | 9 | 917 |
| *thyA* | 8 | 840 |
| *ald* | 8 | 498 |
| *embC* | 6 | 432 |
| *mmpR5* | 4 | 329 |
| *rplC* | 3 | 246 |
| *ddn* | 3 | 192 |
| *panD* | 3 | 146 |
| *rrl* | 3 | 83 |
| *kasA* | 2 | 81 |
| *embR* | 2 | 68 |
| *fbiA* | 2 | 33 |
| *rpsA* | 1 | 13 |
| *ribD* | 1 | 9 |
| *fgd1* | 1 | 3 |

Mutation Count: count of mutation counted across the 68k dataset samples, Mutation Frequency: frequency of mutation of 68k samples in each gene.

**S2 Table**

**Amplicons and genes covered**

| **Amplicon ID** | **Gene** | **Drug** | **No. mutations*** | **Median Portugal_MDR coverage**** | **Median**  **Angola_MDR coverage***** |
| --- | --- | --- | --- | --- | --- |
| A1-mb | *rpoB* | Rifampicin | 160 | 1693 | 1969 |
| A1-gb | *katG* | Isoniazid | 116 | 7148 | 9642 |
| A2-gb | *katG* | Isoniazid | 296 | 972 | 1052 |
| A2-mb | *pncA* | Pyrazinamide | 961 | 5119 | 6681 |
| A3-gb | *katG* | Isoniazid | 308 | 987 | 1145 |
| A3-mb | *embB* | Ethambutol | 89 | 678 | 745 |
| A4-gb | *mmpR5* | Bedaquiline | 6 | 2257 | 3147 |
| A4-mb | *rpsL* | Streptomycin | 11 | 373 | 477 |
| A5-mb | *fabG1* | Isoniazid | 9 | 1269 | 1479 |
| A6-mb | *ddn* | Pretomanid | 3 | 1852 | 2045 |
| A7-mb | *rrl* | Linezolid | 2 | 1328 | 1204 |
| A8-mb | *gyrB* | Fluoroquinolones | 17 | 3687 | 5197 |
| A9-mb | *rrs* | Aminoglycosides | 6 | 1144 | 1038 |
| A10-mb | *gid* | Streptomycin | 136 | 98 | 62 |
| A11-mb | *rplC* | Linezolid | 1 | 2461 | 3190 |
| A12-mb | *rrs* | Aminoglycosides | 11 | 1016 | 1083 |
| A13-mb | *eis* | Kanamycin | 10 | 3934 | 5428 |
| A14-mb | *ethA* | Ethionamide | 357 | 4359 | 5642 |
| A15-mb | *ethA* | Ethionamide | 98 | 2885 | 4511 |
| A16-mb | *embA* | Ethambutol | 6 | 4722 | 5688 |
| A17-mb | *inhA* | Isoniazid | 7 | 2488 | 3814 |
| A18-mb | *ethA* | Ethionamide | 242 | 11324 | 18630 |
| A19-mb | *ahpC* | Isoniazid | 14 | 79 | 195 |
| A20-mb | *folC* | PAS | 16 | 5179 | 7366 |
| A21-mb | *gyrB* | Fluoroquinolone | 24 | 6582 | 8503 |
| A22-mb | *embB* | Ethambutol | 4 | 10291 | 12330 |
| A23-mb | *ethA* | Ethionamide | 83 | 8156 | 10618 |
| A24-mb | *alr* | D-cycloserine | 2 | 19676 | 26800 |
| A25-mb | *thyX* | PAS | 1 | 8994 | 11694 |
| A26-mb | *tlyA* | Capreomycin | 149 | 8532 | 12413 |
| A27-mb | *thyA* | PAS | 8 | 7020 | 10617 |
| A28-mb | *rpoB* | Rifampicin | 1 | 14252 | 21415 |
| A29-mb | *thyA* | PAS | 11 | 22726 | 28514 |

PAS Para-aminosalicylic acid; * in the 68k dataset; ** ONT sequencing of sequencing of the Clinical Portuguese strain; *** ONT sequencing of the Clinical Angola strain

**S3 Table**

**Coverage of the 33 amplicons of the 68k database unique drug resistance mutation**

| **Drug** | **No. detected unique mutations** | **68k database unique mutation** | **% covered** |
| --- | --- | --- | --- |
| ethionamide | 1069 | 1078 | 99.2 |
| pyrazinamide | 987 | 1140 | 86.6 |
| isoniazid | 851 | 865 | 98.4 |
| capreomycin | 174 | 301 | 57.8 |
| streptomycin | 166 | 167 | 99.4 |
| rifampicin | 165 | 168 | 98.2 |
| ethambutol | 99 | 139 | 71.2 |
| ciprofloxacin | 41 | 41 | 100.0 |
| fluoroquinolones | 41 | 41 | 100.0 |
| levofloxacin | 41 | 41 | 100.0 |
| moxifloxacin | 41 | 41 | 100.0 |
| ofloxacin | 41 | 41 | 100.0 |
| para-aminosalicylic_acid | 36 | 37 | 97.3 |
| kanamycin | 16 | 16 | 100.0 |
| amikacin | 7 | 7 | 100.0 |
| clofazimine | 6 | 6 | 100.0 |
| bedaquiline | 6 | 6 | 100.0 |
| aminoglycosides | 6 | 6 | 100.0 |
| linezolid | 3 | 3 | 100.0 |
| delamanid | 3 | 8 | 37.5 |
| cycloserine | 2 | 15 | 13.3 |

**S4 Figure**

**Coverage of mutations identified in the candidate genes in the 68k dataset across the number of amplicons**

**
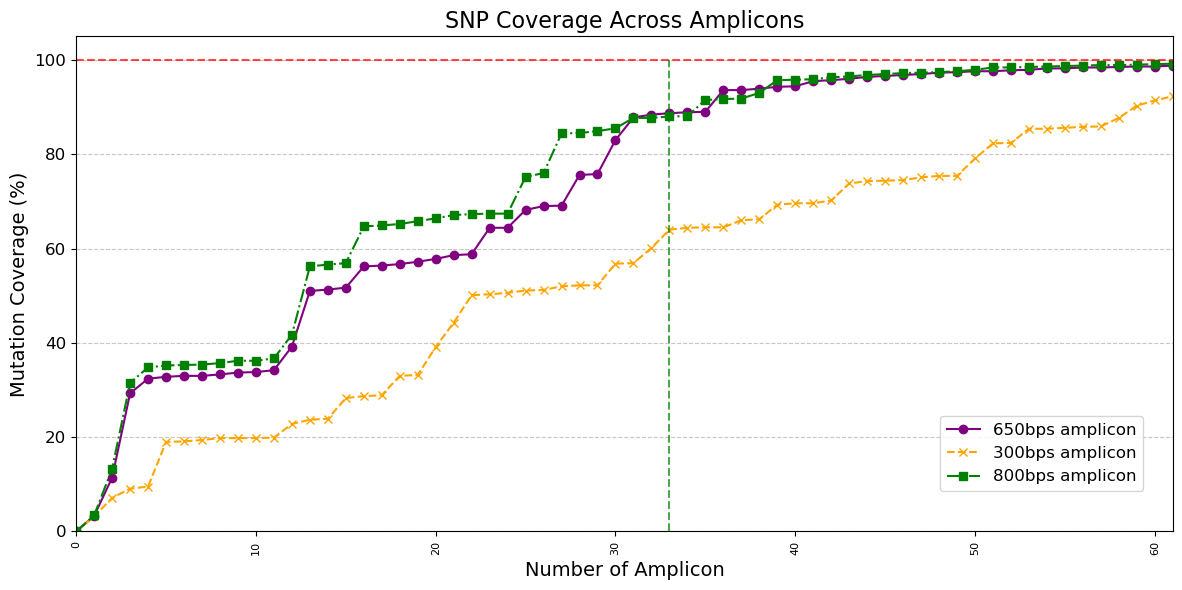
**

Red dashed line: 100% mutation coverage threshold. Green dashed line: 33 amplicons

**S5 Figure**

**The distribution of coverage depth across amplicon sizes**

Depth


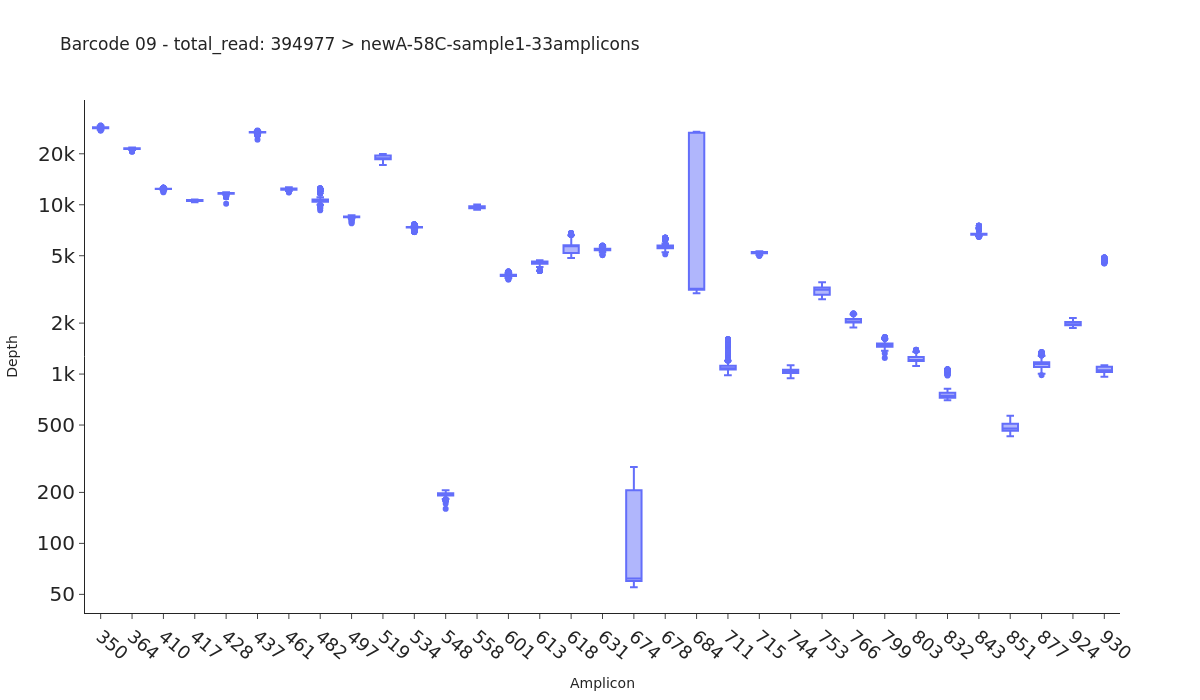


Amplicon sizes (bps)

**S6 Figure**

**Boxplots of ONT sequence coverage depth for the 33 amplicons at 2 hour time point**

1. **Clinical Portuguese strain (Portugal_MDR)**


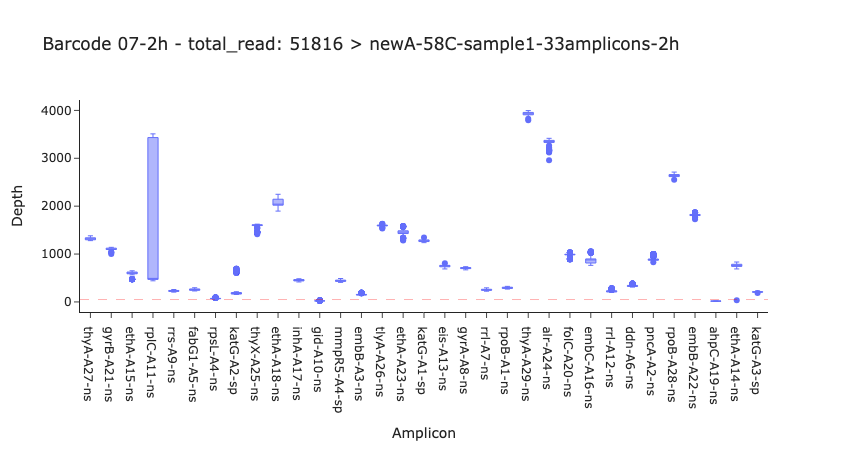

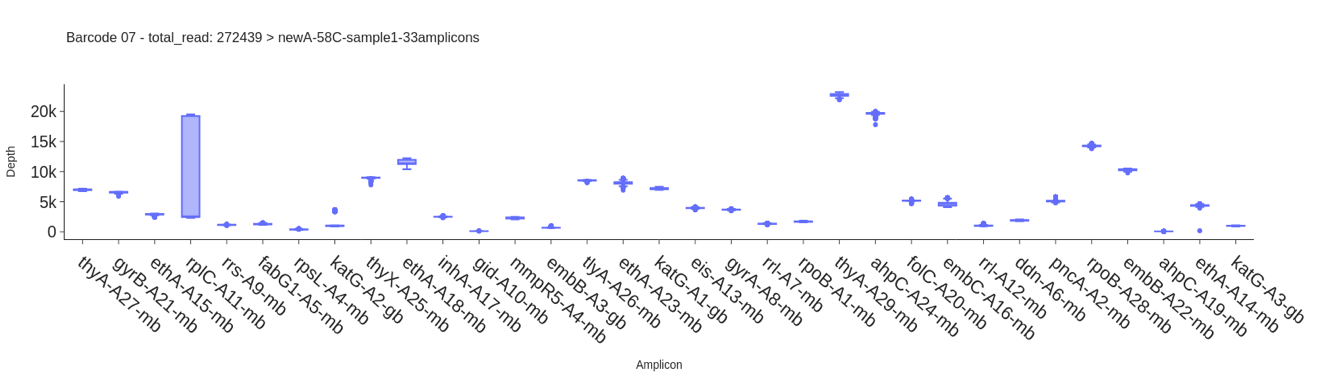


1. **Clinical Angola strain (Angola_MDR)**


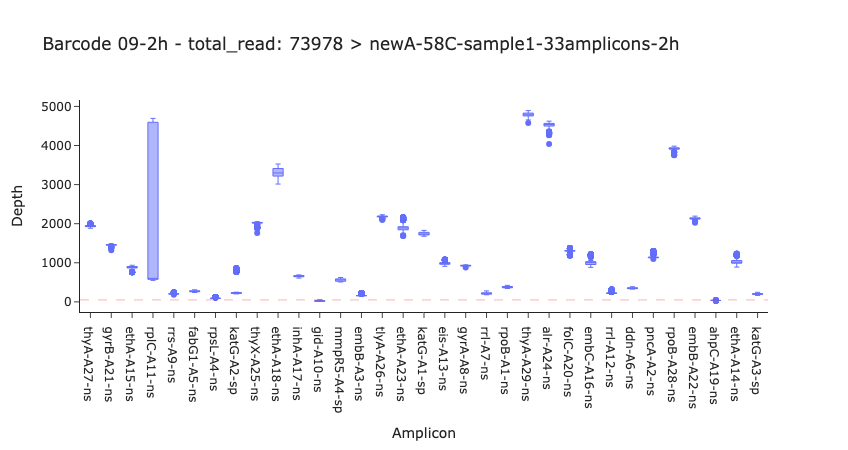

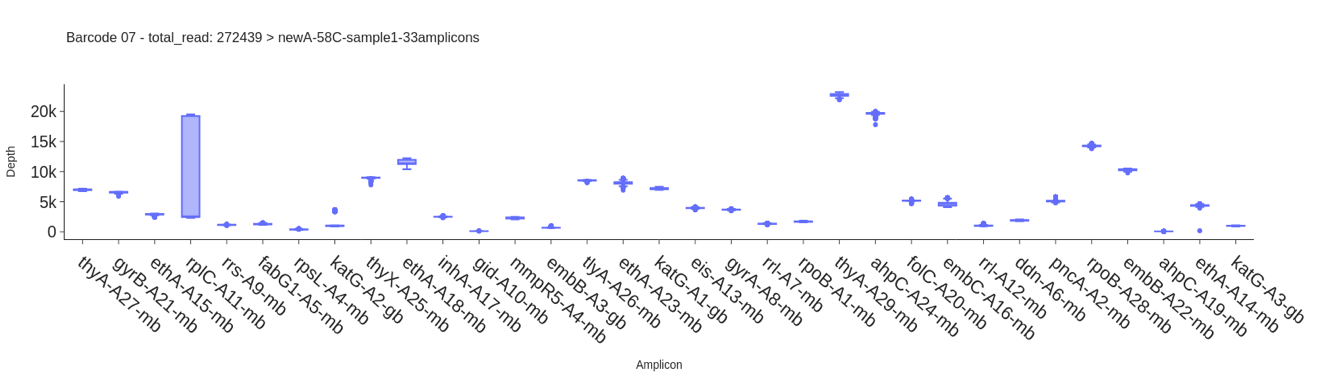


Dashed red line marks a 50-fold coverage threshold
